# SCF/c-KIT Signaling Increased Mucin2 Production by Maintaining Atoh1 Expression in Mucinous Colorectal Adenocarcinoma

**DOI:** 10.3390/ijms19051541

**Published:** 2018-05-22

**Authors:** Ping Shen, Shu Yang, Haimei Sun, Guilan Li, Bo Wu, Fengqing Ji, Tingyi Sun, Deshan Zhou

**Affiliations:** 1 Department of Histology and Embryology, School of Basic Medical Sciences, Capital Medical University, Beijing 100069, China; shenping2014@sina.com (P.S.); yangshu@ccmu.edu.cn (S.Y.); haimei@ccmu.edu.cn (H.S.); lancylee222@163.com (G.L.); wubo2007@ccmu.edu.cn (B.W.); jfq@ccmu.edu.cn (F.J.); styj211@ccmu.edu.cn (T.S.); 2 Beijing Key Laboratory of Cancer Invasion and Metastasis Research, Beijing 100069, China; 3 Cancer Institute of Capital Medical University, Beijing 100069, China

**Keywords:** Atonal homologue 1 (Atoh1), c-KIT, colorectal cancer, Mucin2, mucinous colorectal adenocarcinoma

## Abstract

Mucinous colorectal adenocarcinoma (MCA) patients often a show high risk of malignant potential and a poorer survival rate. Given that the pathological feature and oncobiological characteristics of MCA are correlated with its abundant extracellular mucin2 (MUC2), we paid interest toward investigating the key factor that promotes MUC2 production exposure to highly-activated stem cell factor (SCF)/c-KIT signaling, which we believed to contribute to MCA formation. Long-term azoxymethane and dextran sodium sulfate treatment successfully induced MCA only in wild-type (WT) mice at week 37 and 43, while all *c-kit* loss-of-function mutant mice (Wads^m/m^) developed non-MCA. Significantly, MUC2 and its key transcriptional factor Atonal homologue 1 (Atoh1) were remarkably expressed in MCA mice compared with non-MCA mice. Atoh1 was significantly elevated in colorectal cancer (CRC) cells stimulated by exogenous SCF or overexpressing c-KIT in vitro, while decreased by the blockage of SCF/c-KIT signaling with Imatinib. Furthermore, the maintained Atoh1 protein level was due to the inactive glycogen synthase kinase 3β (p-GSK3β) by virtue of the activated SCF/c-KIT-Protein Kinase B (AKT) signaling. Similar results were obtained from the ONCOMINE database and CRC patients. In conclusion, we suggested that SCF/c-KIT signaling promoted MUC2 production and MCA tumorigenesis by maintaining Atoh1 expression. Therefore, targeting the related key molecules might be beneficial for treating MCA patients.

## 1. Introduction

Colorectal cancer (CRC) is the fourth leading cause of cancer-related deaths around the world [1]. Mucinous colorectal adenocarcinoma (MCA), a subtype of CRC accounting for 15–20% of all CRC patients, is defined as carcinoma with more than 50% of extracellular mucinous component [2]. Patients suffering from MCA often show a higher risk of malignancy and poor five-year survival rates compared with regular CRC (non-MCA) [3,4], but the mechanism of MCA development has not been completely elucidated.

MCA is characterized by often occurring in the right colon, microsatellite instability high (MSI-high), high frequency of CpG island methylator phenotype (CIMP-high), and frequent BRAF mutation [5,6,7]. Additionally, recent studies indicated that the receptor tyrosine kinase (RTK) family could contribute a great dael to the development of multiple mucinous cancers [8,9,10]. c-KIT, a type III RTK activated by stem cell factor (SCF), was upregulated in ovarian serous carcinomas and intraductal papillary mucinous pancreatic neoplasm [11,12]. Significantly, c-KIT showed strong activity in 14% of primary CRC patients and 18% of primary tumors and xenografts, who often displayed a stronger capacity of proliferation, a higher risk of metastasis, and a poorer prognosis [13,14]. Our previous results showed that azoxymethane (AOM) and dextran sodium sulfate (DSS) induced MCA that occurred in wild-type (WT) mice at week 37, while *c-kit* loss-of-function mutant mice (Wads^m/m^) only developed regular CRC (non-MCA) [15], preliminarily indicating that c-KIT signaling played a key role in the development of MCA.

Based on pathological features, the production of massive amounts of mucus is a pivotal process during MCA development. Mucin2 (MUC2), the well-known mucus component in a large number of mucinous cancers, is related with the high malignancy and strong invasive ability [16,17,18]; it is well known that the mucinous cancer-related MUC2 is basically the same as that in goblet cells [19]. Hence, it is a feasible way to determine how the mucinous cancer-related MUC2 is upregulated in light of the regulation of MUC2 in goblet cells. Atonal homologue 1 (Atoh1), also known as mouse atonal homolog 1 (*Math1*) gene in mice and human atonal homolog 1 (*Hath1*) gene in humans, is a basic helix-loop-helix transcription factor which not only triggers the differentiation, but also promotes the transcription of MUC2 in goblet cells [19,20,21]. In cancers, Atoh1 was frequently hyper-expressed in mucinous cancers, but rarely in non-mucinous cancers, indicating a possible involvement of Atoh1 in MCA tumorigenesis [22]. It is worth noting that Atoh1 expression could be regulated by RTKs and hence, we wondered whether c-KIT signaling participated in the formation of MCA by regulating Atoh1 expression. Our results demonstrated that activated SCF/c-KIT could elevate Atoh1 expression and promote the formation of MCA. It might be helpful in seeking targeted therapies of MCA and improving the current understanding of other mucinous cancers.

## 2. Results

### 2.1. Mucinous Colorectal Adenocarcinoma (MCA) Was Much More Efficiently Induced in WT Mice than in Wads^m/m^ Mice

**Figure 1:**
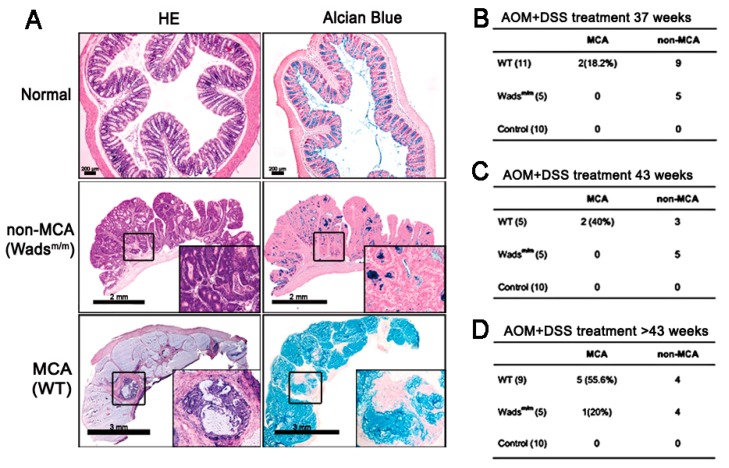
Establishment of the mucinous colorectal adenocarcinoma (MCA) murine model. (**A**) Azoxymethane (AOM) and dextran sodium sulfate (DSS) treatment for >37 weeks successfully induced colorectal cancer (CRC) in both WT and Wads^m/m^ mice. Hematoxylin-eosin (HE) staining showed the typical pathological characteristics of CRC including “back to back” dysplastic glands and abnormal nuclear divisions in all tumors. Additionally, some CRC tissues from WT mice exhibited the MCA subtype, as indicated by massive Alcian Blue-positive mucus. High magnification views are in the insets of each frame. Normal saline administered mice were set as normal controls. (**B**–**D**) The incidence of MCA in WT and Wads^m/m^ mice at the indicated sacrifice time.

In the first place, we established the MCA murine model using *c-kit* loss-of-functional Wads^m/m^ mice and WT littermates by long-term AOM + DSS treatment as previous (Figure 1A). At week 37, part of the WT mice (18.2%) developed MCA characterized by the abundant extracellular mucus (Figure 1A,B). When extending the induction time, 40% and 55.6% of the WT mice had MCA at week 43 and beyond, respectively (Figure 1C,D), whereas, except 1 MCA at >43 weeks in the Wads^m/m^ mice, there was no or few mucus in CRC tissues which were defined as non-MCA (Figure 1D). These results implied that intact c-KIT was involved in the MCA phenotype.

### 2.2. Highly-Activated c-KIT Signaling Promoted Mucin 2 (MUC2) Expression via Increasing Atonal Homologue 1 (Atoh1)

**Figure 2:**
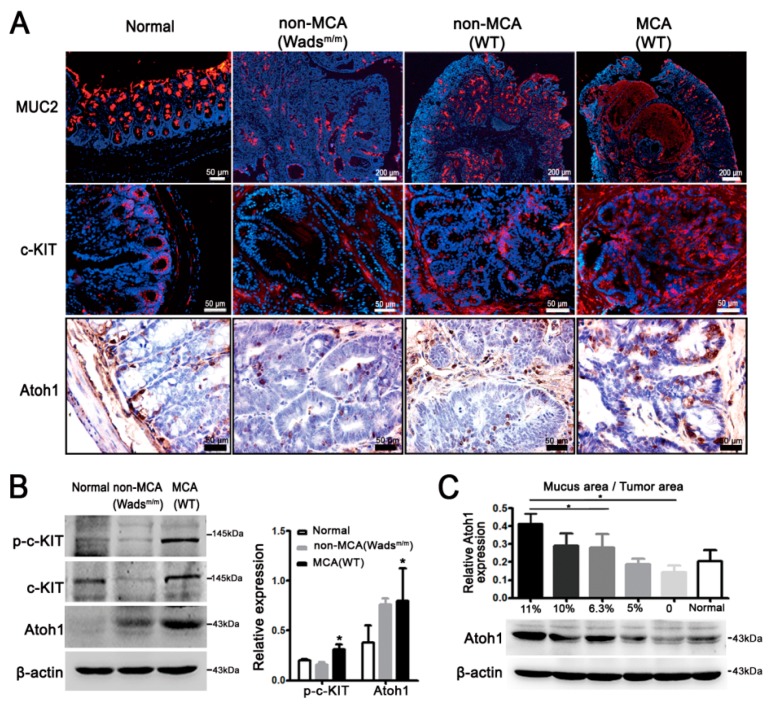
c-KIT increased mucin2 (MUC2) and Atonal homologue 1 (Atoh1) expressions in mice. (**A**) MUC2 was primarily expressed in goblet cells of normal colon and occupied the extracellular mucus in WT-MCA tissues, while very few MUC2 were detected in non-MCA of WT and Wads^m/m^ mice. C-KIT and Atoh1 were located in the basal crypts of normal colon and had more potent immunostaining intensities in MCA compared with non-MCA; (**B**) Western blot showed higher expressions of c-KIT, p-c-KIT, and Atoh1 in MCA than those in non-MCA; * *p* < 0.05; and (**C**) Atoh1 was gradually increased with the increase in the mucus area, * *p* < 0.05. All values are mean ± SEM of three independent experiments unless otherwise stated.

Given that the phenotype and oncobiological characteristics of MCA are correlated with its abundant extracellular MUC2, we then investigated the potential role of c-KIT signaling in MUC2 production. MUC2 covered the cytoplasm of goblet cells in normal colon and extracellular mucus in MCA (Figure 2A). However, there were much less MUC2 in non-MCA tissues from either WT or Wads^m/m^ mice (Figure 2A). In normal colon epithelium, c-KIT, and Atoh1, the determining transcriptional factor for MUC2 was fundamentally expressed in the basal crypts (Figure 2A). What really mattered was that c-KIT and Atoh1 were intensively expressed in MCA from WT mice compared with a weak expression in non-MCA from the WT and Wads^m/m^ mice (Figure 2A). Since there were no significant differences in MUC2, c-KIT, and Atoh1 expressions between WT non-MCA and Wads^m/m^ non-MCA tissues, in the following results, only Wads^m/m^ non-MCA tissues were displayed. Paralleled with staining, activated c-KIT (p-c-KIT), c-KIT, and Atoh1 expression were obviously elevated in MCA compared with non-MCA (*p* < 0.05; Figure 2B). We also noted that the Atoh1 level was gradually increased along with the increase of mucus area (*p* < 0.05; Figure 2C). In addition, Atoh1 was hyper-expressed in WT mice that had higher activity of c-KIT than Wads^m/m^ mice (Figure S2A). These results suggested that highly-activated c-KIT signaling contributed to the MUC2 accumulation, probably via increasing Atoh1.

### 2.3. Stem Cell Factor (SCF)/c-KIT Signaling Increased Atoh1 via Protein Kinase B (AKT) and c-Jun N-Terminal Kinase (JNK) Pathway In Vitro

**Figure 3:**
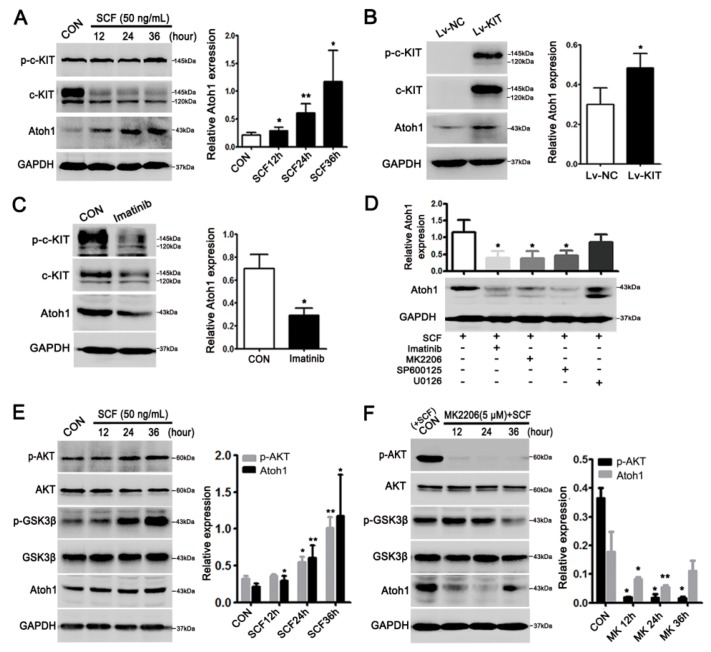
Stem cell factor (SCF)/c-KIT signaling upregulated Atoh1 via Protein Kinase B (AKT) and c-Jun N-terminal kinase (JNK) pathways in LS174T cell. (**A**) Activation of c-KIT by exogenous recombinant human stem cell factor (rhSCF) (50 ng/mL) treatment enhanced Atoh1 expression in a time-dependent manner, * *p* < 0.05, ** *p* < 0.01; (**B**) overexpression of c-KIT by lentivirus encoding *c-kit* clearly upregulated Atoh1 in the presence of rhSCF. Lv-NC, lentivirus-control; Lv-KIT, lentivirus-*c-kit*; * *p* < 0.05 (**C**) Imatinib treatment (2 μM) for 24 h to inhibit SCF/c-KIT signaling evidently attenuated Atoh1 expression, * *p* < 0.05; (**D**) CRC cells were exposed to rhSCF alone or in combination with MK2206 (AKT inhibitor), SP600125 (JNK inhibitor), or U0126 (extracellular signal-regulated kinases (ERK) inhibitor). MK2206 or SP600125 treatment could significantly abrogate the rhSCF-induced Atoh1 expression, * *p* < 0.05; (**E**) activated c-KIT-AKT signaling pathway by rhSCF could up-regulate p-GSK3β, which, thereby, increased Atoh1. * *p* < 0.05, ** *p* < 0.01; and (**F**) inhibiting AKT by MK2206 treatment significantly downregulated p-GSK3β and Atoh1. Cells with rhSCF treatment were used as control, * *p* < 0.05, ** *p* < 0.01. All the values are mean ± SEM of three independent experiments unless otherwise stated.

To elucidate whether c-KIT signaling could regulate Atoh1 expression, we carried out in vitro experiments on the CRC cell line. The LS174T cell line was used because it expressed endogenous c-KIT and Atoh1 and exhibited the goblet cell phenotype. Upon the treatment of recombinant human stem cell factor (rhSCF), Atoh1 was upregulated in a time-dependent manner (*p* < 0.05 or 0.01; Figure 3A). Overexpression of c-KIT also clearly increased Atoh1 in the presence of exogenous rhSCF (*p* < 0.05; Figure 3B). In contrast, blockage of SCF/c-KIT signaling by Imatinib resulted in decreased Atoh1 in CRC cells (*p* < 0.05; Figure 3C).

In view of the exact downstream molecules of SCF/c-KIT signaling involved in the process, CRC cells were further treated with specific inhibitors of AKT, JNK, and extracellular signal-regulated kinases (ERK) pathways, respectively. The results revealed that inhibition of AKT and JNK rather than ERK pathway significantly decreased Atoh1 expression (*p* < 0.05; Figure 3D). GSK3β, a downstream molecule of AKT, promotes Atoh1 degradation by phosphorylating Atoh1 in human CRC cells [23]. Here, we also demonstrated SCF/c-KIT signaling activated the AKT pathway in a time-dependent manner, which led to the inactivation (phosphorylation) of GSK3β and subsequent maintenance of Atoh1 level in CRC cells (*p* < 0.05 or 0.01; Figure 3E). On the contrary, inhibition of the AKT pathway by MK2206 significantly decreased p-GSK3β and Atoh1 level in a time-dependent manner (*p* < 0.05 or 0.01; Figure 3F). Moreover we detected the expressions of AKT and GSK3β in murine models and found that with activated AKT in MCA, GSK3β was obviously inactivated, as indicated by the increasing level of its phosphorylated form (p-GSK3β) (*p* < 0.05; Figure S2B).

### 2.4. c-KIT and Atoh1 Were Increased in Human MCA Tissues

**Figure 4:**
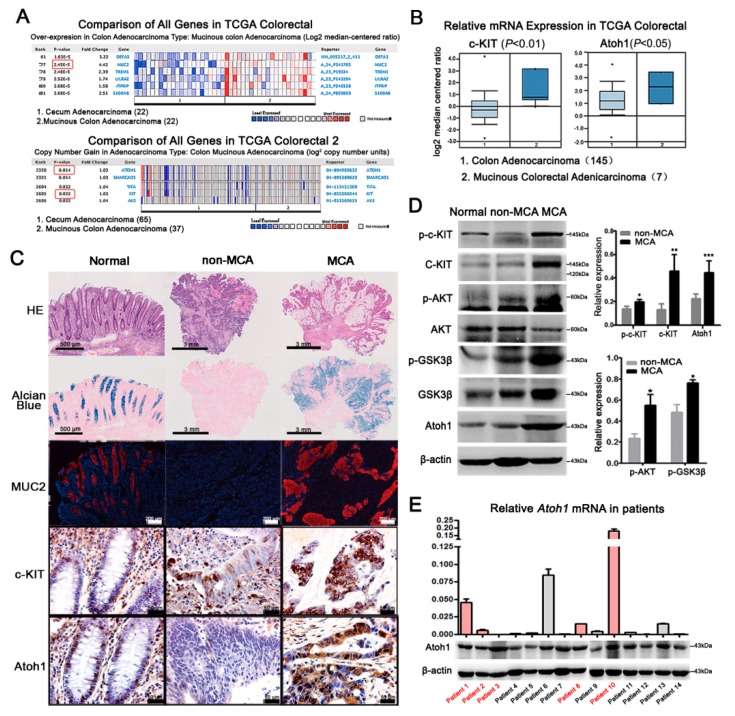
Expressions of c-KIT and Atoh1 in CRC patients. (**A**) Compared with cecum adenocacinoma (*n =* 22), mRNA levels of *MUC2* was markedly increased in mucinous colon adenocarcinoma (*n =* 22) in TCGA Colorectal (red boxes, *p*-value). DNA copies of *c-KIT* and *Atoh1* were significantly increased in mucinous colon adenocarcinoma (*n =* 37) indicated by TCGA Colorectal 2; (**B**) the mRNA levels of *c-KIT* and *Atoh1* were also elevated in MCA patients (*n =* 145) compared with non-MCA (*n =* 7) from TCGA Colorectal. *p* < 0.05 or 0.01; (**C**) according to the mucus area estimated under HE and Alcian blue staining, human CRC tissues were divided into non-MCA and MCA. Paratumoral normal tissues were used as controls. MUC2 was filled in goblet cells in normal colon and the main mucus component in MCA, while there was rare MUC2 in non-MCA tissues. Normally, c-KIT was expressed in the membrane while Atoh1 in the nuclei of epithelial cells, including goblet cells. Compared with non-MCA, c-KIT and Atoh1 were remarkably elevated in MCA, indicated by much more intensive immunostaining; (**D**) protein expressions of total c-KIT, p-c-KIT, and Atoh1 were clearly higher in MCA patients (*n =* 5) than those in non-MCA patients (*n =* 9) combined with an increased expression of p-AKT and p-GSK3β. * *p* < 0.05, ** *p* < 0.01, *** *p* < 0.001; and (**E**) real-time PCR was performed to detect *Atoh1* mRNA level in CRC tissues from 14 patients, including 5 MCA patients marked by red color. *Glyceraldehyde-3-phosphate dehydrogenase* (GAPDH) was used as the internal control. Normalized *Atoh1* mRNA expression was shown in the column graph. Western blot showed the protein expression of Atoh1 which was consistent with its mRNA level except patient 3. All values are mean ± SEM of three independent experiments unless otherwise stated.

Finally, we verified our results in the ONCOMINE database and CRC patients. The ONCOMINE database showed that *MUC2* was listed in the forefront of genes whose expressions were clearly upregulated in MCA cohorts compared with non-MCA (*p* < 0.01; Figure 4A). Similarly, DNA copies and mRNA levels of *c-KIT* and *Atoh1* were significantly higher in MCA than those in non-MCA (*p* < 0.05 or 0.01, Figure 4A,B). Therefore, we collected eight MCA and 16 non-MCA patients defined by hematoxylin-eosin (HE) and Alcian blue staining (Figure 4C), clinical documents suggested that MCA tended to have higher frequency of lymphatic invasion and distant metastasis, suggesting a worse outcome compared with non-MCA patients (Table S1). Consistent with our results obtained from the murine model, MUC2 covered the extracellular mucus in MCA combined with an increased expression of c-KIT and Atoh1 (Figure 4C). Next, we checked the expression of related molecules in 14 fresh tumor samples, which include five MCAs and nine non-MCAs. c-KIT and its downstream AKT-GSK3β pathway were highly activated in MCA patients compared with non-MCA (*p* < 0.05 or 0.001; Figure 4D). Thereupon, *Atoh1* mRNA and protein levels in MCA patients were significantly higher than non-MCA patients (Figure 4E). These results suggested that c-KIT upregulated Atoh1 accelerated the differentiation of mucus-producing tumor cells and MUC2 production during the development of MCA.

## 3. Discussion

Accumulating evidence suggests that CRCs are a group of heterogeneous diseases at the molecular level that are characterized by a range of genomic and epigenomic alterations and can also be classified into several subtypes by integrated molecular characterization (TCGA Classification), CRC gene expression profiling (CMS classification), and key molecular features (five subtypes) [24]. It was reported that MCA constitutes a distinct subentity of CRC and may be classified into CMS 1 or Type 1, which is characterized as showing MSI-high, CIMP-high, and frequent BRAF mutation; and commonly seen in older and female patients in the proximal colon [25,26]. However, the underlying mechanism of its development has not been completely understood. In the present study, we identified a significant relevance between SCF/c-KIT signaling and MCA formation using a murine model, CRC cell line, and human samples. We demonstrated that highly-activated SCF/c-KIT-AKT signaling promoted MUC2 production and consequent MCA phenotype by increasing Atoh1 expression.

We found that long-term treatment by AOM + DSS efficiently induced the MCA phenotype in WT mice rather than c-kit loss-function mutant Wads^m/m^ mice, indicating that c-KIT signaling might facilitate MCA phenotype.

MUC2 and MUC5AC were the main proteins significantly increased in MCA. The *MUC2* gene encodes for a typical secretory mucin, which is predominantly found in colorectal goblet cells and cancerous mucus; while the *MUC5AC* gene is mainly expressed in the gastric epithelium, thereby, the expression of MUC5AC in MCA may imply gastric differentiation [17]. However, MUC5AC often showed co-expression of MUC2, indicating that the cancer still has goblet cell lineage, and its gastric differentiation may simply be superficial [17]. Large amounts of MUC2 in MCA endowed the tumor cells with higher malignancy than non-MCA [18]. Targeting MUC2 as a novel therapeutic strategy has been reported to inhibit mucinous tumor growth and improve survival in a murine xenograft model of pseudomyxoma peritonei [27]. In MCA mice, p-c-KIT was significantly increased with large amount of MUC2, confirming the role of SCF/c-KIT signaling in promoting MUC2 production in MCA. It should be noted that our mice showed a higher incidence of MCA (over 50%) that might be attributed to the DSS-induced chronic inflammation that promoted the occurrence of MCA. When extending the induction time beyond 43 weeks, one Wads^m/m^ mice developed MCA, indicating that any other factors probably participated in the process of MCA formation.

During gastrointestinal epithelia development, Atoh1 transcription factor promoted precursor cells of the epithelium to differentiate into goblet cells and up-regulated MUC2 expression via binding to the MUC2 promoter region [19,20,21]. Furthermore, transfection of the *Atoh1* gene alone could not induce the mucinous phenotypic gene expression because of proteasomal degradation of the Atoh1 protein. In contrast, treatment with tumor necrosis factor-α (TNF-α) induced stable expression of Atoh1 that resulted in the acquisition of the characteristic of mucinous cancer [28]. Moreover, Atoh1 induced a higher malignant potential rather than the differentiation phenotype of mucinous cancer via enhancing the enrichment of cancer stem cells [29]; and, therefore, highly-expressed Atoh1 in tumor tissues might be the reason for MCA formation and why MCA has a more malignant potential than non-MCA does. Others have reported that CDX2 (a caudal-related homeobox transcription factor) and Spdef (SAM pointed domain containing ETS transcription factor) were also implicated in mucus production and goblet cell differentiation [30,31]. CDX2 could induce expression of Atoh1 and promote differentiation of epithelial stem cells into Atoh1-positive multipotent intermediate cells, thereby leading to produce the secretory cell lineages [32], while Spdef functioned as a downstream molecule of Atoh1, which was a key transcription factor for the terminal differentiation and maturation of goblet cells [33]. Thus, Atoh1 was the determinant in the early stage of secretory cell determination and in the MUC2 production of the intestinal epithelium and MCA.

In the present study, we observed that Atoh1 was expressed in the basal crypt of normal colon where the most intestinal stem cells and goblet cells usually are. In particular, c-KIT, which was known as a marker of intestinal stem cells, also existed in this area and in goblet cell [14]. Furthermore, there was an increased expression of c-KIT in Atoh1-positive cells [34]. This indicated that SCF/c-KIT signaling might be involved in the differentiation of the precursor cells into goblet cells by upregulating Atoh1 expression in the development of normal epithelium. The expressions of c-KIT and Atoh1 were simultaneously elevated in MCA compared with non-MCA specimens from mice and patients. Additionally, the in vitro experiments added to the evidence that SCF/c-KIT increased Atoh1 expression, which further induced MUC2 transcription and the MCA phenotype. We further observed that activated JNK and AKT pathways by SCF/c-KIT signaling were relevant to the upregulation of Atoh1 in vitro. It has been suggested that Atoh1 mRNA transcription was facilitated by JNK in normal intestinal epithelium [35], whereas Atoh1 expression was mainly regulated via post-translational modification in CRC development and the activation of AKT could induce the inactivation of GSK3β, which was involved in the ubiquitin-proteasomal degradation of Atoh1 in CRC cells [23,28]. Similarly, we found increased Atoh1 accompanied by highly-activated SCF/c-KIT-AKT signaling and inactivated GSK3β in MCA mice and patients, supporting the theory that SCF/c-KIT signaling protected Atoh1 from proteasomal degradation induced by AKT-GSK3β in the pathogenesis of MCA.

We also noticed that there was no apparent difference in the number of colonic goblet cells between the WT and Wads^m/m^ mice, except more mucus was attached to the epithelial surface in the WT mice (Figure S2C,D), in light of which we presumed that, apart from promoting MUC2 synthesis, SCF/c-KIT signaling might also accelerate the secretion of MUC2.

In summary, the present study provided a new insight into the pathogenesis of MCA. SCF/c-KIT signaling and its downstream AKT-GSK3β pathway promoted MCA formation by enhancing the level of Atoh1 protein, which induced the production of massive mucin. Therefore, the SCF/c-KIT-AKT-GSK3β-Atoh1 axis might be a potential therapeutic target for treating MCA in the future.

## 4. Materials and Methods 

### 4.1. Animals and Ethics Statement

Heterozygous C57BL/6J mice with *c-kit* loss-of-function mutant (Wads^m/+^) were purchased from the Model Animal Research Center of Nanjing University (Nanjing, China). Homozygous mice (Wads^m/m^) were generated by intercross of Wads^m/+^ mice. Wild-type (WT) C57BL/6J mice were used as the control. Mice (4 mice/cage) were maintained in a specified pathogen-free (SPF) environment with controlled conditions of humidity (50 ± 10%), light (12/12-h light/dark cycle), and temperature (23 ± 2 °C). Animal studies were carried out strictly under protocols approved by the Animal Care and Use Committee of Capital Medical University (permit number AEEI-2014-058, 17 June 2014). Every effort was made to minimize the number of mice used as well as their suffering.

### 4.2. MCA Murine Model

The MCA murine model was established as previously described. In brief, each mouse was intraperitoneally injected with azoxymethane (AOM, 10 mg/kg; Sigma-Aldrich, St. Louis, MO, USA) once, followed by three periods of intermittent 2.5% dextran sodium sulfate (DSS; MP Biomedicals, Rosemont, IL, USA) in water. Control mice were injected with normal saline and had free access to water. Our previous studies showed that MCA was successfully induced 37 weeks after AOM injection. In this study, the mice were sacrificed at week 37, week 43, and beyond respectively.

### 4.3. Patients

Twenty-four pairs of human specimens, including CRC tissues and paratumorous normal tissues, were obtained from the department of gastrointestinal surgery, Beijing Friendship Hospital, Capital Medical University. All procedures involving human specimens were performed with written informed consent according to the Declaration of Helsinki, and the research was approved by the Ethics Committee of Capital Medical University (Permit Number 2015SY12, 9 March 2015). The CRC specimens were classified into MCA and non-MCA according to the amount of mucus defined by Alcian blue staining. Hematoxylin-eosin (HE) staining was used for oncopathological examination.

### 4.4. Cell Culture

The human CRC-derived LS174T cell line was purchased from American Type Culture Collection (ATCC) and cultured in Dulbecco’s modified Eagle medium (DMEM; Gibco, YN, USA) supplemented with 10% fetal bovine serum (FBS; Invitrogen, Carlsbad, CA, USA) and 1% penicillin/streptomycin (Gibco) at 37 °C under a humidified atmosphere of 5% CO_2_ and 95% air. Cells were treated with recombinant human SCF (rhSCF) (50 ng/mL; R&D System, Minneapolis, MN, USA) for 12, 24, or 36 h in order to activate c-KIT, While Imatinib (2 μM; BioVision, Zurich, Switzerland) was used to block SCF/c-KIT signaling activity. After serum starvation overnight, AKT, JNK, and ERK signaling was inhibited by MK2206 2HCL (5 μM; Selleck Chemicals, Shanghai, China), SP600125 (10 μM; Sigma-Aldrich), U0126 (10 μM; Sigma-Aldrich respectively), and rhSCF (50 ng/mL) was added 1 h after inhibitor treatment. Cells were harvested 24 h later.

### 4.5. Cryosectioning and Immunofluorescent Staining 

Cryosections (7 μm) were cut using a cryostat (Leica CM3050S, Leica, Wetzlar, Germany) and fixed with 100% acetone or 4% paraformaldehyde (PFA) for 30 min at 25 °C. Nonspecific binding sites were blocked with 1% bovine serum albumin (BSA; Sigma-Aldrich) for 1 h, then sections were incubated with anti-MUC2 (H-300, 1:200; Santa Cruz Biotechnology, Santa Cruz, CA, USA) or anti-mouse c-KIT (CD117, 1:300; eBioscience, San Diego, CA, USA) antibody at 4 °C overnight followed by incubation with corresponding Cy3-conjugated secondary antibody for 1 h at 25 °C in the dark. Sections were mounted with mounting medium with DAPI (Zhongshanjinqiao Biotechnology, Beijing, China) and visualized by a fluorescence microscope (Nikon, Eclipse Ni, Tokyo, Japan).

### 4.6. Paraffin-Sectioning and Immunohistochemical Staining

One-hundred percent acetone or 4% PFA fixed tissues were embedded in paraffin and sectioned (5 μm) using a paraffin microtome (Leica RM 2135, Leica, Germany). The endogenous peroxidase activity was blocked by 0.3% H_2_O_2_/Methanol for 15 min. Sections were incubated with anti-Math1 (M-156, 1:200; Santa Cruz Biotechnology, Santa Cruz, CA, USA) or anti-human c-KIT (CD117, 1:200; Dako, Glostrup, Denmark) rabbit polyclonal antibody at 4 °C overnight followed by incubation with corresponding horseradish peroxidase (HRP)-conjugated secondary antibody (Zhongshanjinqiao Biotechnology, Beijing, China) at 25 °C for 45 min. Reactions were visualized by H_2_O_2_/DAB solution and counterstained with hematoxylin and observed by a light microscope (Nikon, Eclipse Ni, Tokyo, Japan).

### 4.7. Alcian Blue/Periodic Acid-Schiff (PAS) Staining

Four percent PFA fixed and paraffin-embedded tissues were cut and stained with Alcian blue in the traditional way. The mucus area was picked up by “quick select” and recorded in dpi by Photoshop (Photoshop CS3 Extented, Adobe, San Jose, CA, USA). The distal colon of mice were removed and immediately submerged in Methanol-Carnoy’s fixative at 4 °C for 3 h followed by 100% methanol for 30 min, 100% ethanol for 20 min, and xylene for 15 min. Fixed tissues were embedded in paraffin and cut into 7 μm sections using a paraffin microtome. Sections were stained with Alcian blue/PAS.

### 4.8. Western Blot

Total proteins were extracted using RIPA lysis buffer containing protease inhibitor (Applygen, Beijing, China) and phosphatase inhibitor (Sigma-Aldrich). Ten percent SDS-PAGE (sodium dodecyl sulfate polyacrylamide gel electrophoresis) was performed and proteins were transferred to PVDF membranes (Millipore, Burlington, MA, USA). After blocking with Tris-buffered saline containing 0.05% Tween-20 (TBST) and 5% non-fat dry milk or 5% BSA for 1 h, membranes were incubated with the following primary antibodies including anti-c-KIT (C-19, 1:600; Santa Cruz, CA, USA), anti-Atoh1 (1:600; Proteintech, Chicago, IL, USA), anti-Phos-c-KIT (Tyr719) (1:1000, Cell Signaling Technology, Danvers, MA, USA), anti-AKT (pan) (1:1000, Cell Signaling Technology), anti-Phos-AKT (Ser473) (1:1000, Cell Signaling Technology), anti-GSK-3β (1:1000, Cell Signaling Technology), and anti-Phos-GSK-3β (Ser9) (1:1000, Cell Signaling Technology) antibodies. Then, membranes were incubated with corresponding HRP-conjugated secondary antibodies (Abcam, Cambridge, MA, USA) for 1 h at 25 °C. A rabbit anti-GAPDH or anti-β-actin (Cell Signaling Technology) antibody was used as an internal control. Proteins were detected with enhanced chemiluminescence (ECL) (ThermoFisher Scientific, Waltham, MA, USA) and their intensity was analyzed with Image J software (ImageJ2X, NIH, Bethesda, MD, USA).

### 4.9. Overexpression of c-KIT

c-KIT was overexpressed in vitro in LS174T cells mediated by lentivirus with the technical support from GeneChem (Shanghai, China). Cells were seeded in six-well plates at a density of 5 × 10^4^ cells/well and infected with Lv-*c-kit* (GV287, GeneChem; Figure S1) when reaching 30% confluence. Three days later, infection efficiency was evaluated by observing GFP expression by using an inverted fluorescence microscope (Leica DMI3000B, Leica). Total proteins were extracted and analyzed as previously described.

### 4.10. RNA Exraction and Real-Time PCR

Total RNA was extracted from 14 fresh samples of CRC patients with TRIzol reagent (Life Technologies, Carlsbad, CA, USA). RNA concentration was measured using a Nanodrop 2000c spectrophotometer (Thermo Scientific, Waltham, MA, USA). The 260/280 absorbance ratio of RNA ranged from 1.8 to 2.0. Formaldehyde denaturing gel electrophoresis showed clear 28S and 18S rRNA bands, and the 8S rRNA band was approximately twice that of the 18S rRNA band, indicating an intact and usable RNA. Reverse transcription reactions were performed using a cDNA Reverse Transcription Kit (Applied Biological Materials, Richmond, BC, Canada). The total reaction mixture (20 μL) contained 2 μL (1–1.5 μg) of total RNA, and 4 μL 5× All-in-one RT MasterMix and Nuclease-free H_2_O. The reactions were incubated in a Veriti 96-well thermal cycler (Life Technologies) for 40 min at 42 °C and 5 min at 85 °C. Real-time PCR was performed with an ABI 7500 real-time PCR system using Ultra SYBR Mixture with ROX (Life Technologies). The reactions were incubated at 95 °C for 10 min, followed by 40 cycles of 95 °C for 15 s and 60 °C for 1 min. We diluted the template into a series of concentration gradients for the PCR reaction and standard curve. The amplification efficiency calculated by the software was about 95%, within the valid range (90–110%). The primers of Atoh1 were designed by using NCBI’s Primer-BLAST software in Pubmed, and spanned exon-exon junctions and, therefore, can specifically bind to mRNA (forward: AGAGAGCATCCCGTCTACCC; reverse: TTCTGCACCCCATTCACCTG). According to the research reports, GAPDH was used as the internal control (forward: AGAAGGCTGGGGCTCATTTG, reverse: AGGGGCCATCCACAGTCTTC). All reverse transcription reactions included no-template controls. All PCR reactions were run in triplicate. Relative gene expression was determined using the comparative C_T_ (2^−∆∆*C*t^) method.

### 4.11. ONCOMINE Database Analysis

The DNA copies and mRNA levels of MUC2, c-KIT and Atoh1 in CRC patients were analyzed in the ONCOMINE database (http://www.oncomine.org), a cancer microarray database and web-based data-mining platform that combines the databases of the Cancer Genome Atlas (TCGA) (https://cancergenome.nih.gov/) and other databases. The expression level of genes was compared between the MCA and non-MCA patients among different databases.

### 4.12. Statistics

Continuous variables were presented as the mean ± standard deviation, and categorical variables were presented as the percentages. Continuous variables were analyzed by Student’s *t*-test or one-way ANOVA with the SPSS 17.0 software (IBM Corporation, New York, NY, USA), and the chi-square test was used to compare categorical variables between MCA and non-MCA groups. A *p*-value of 0.05 or less was considered statistically significant. We retrieved data from an open-source de-identified database, The Cancer Imaging Archive (TCIA), which is the imaging counterpart of TCGA. TCGA, in brief, is a coordinated effort led by the National Cancer Institute to accelerate the molecular and genomic understanding of cancer. The TCGA program performs genomic sequencing and characterization of tissues from cancers diagnosed and treated at cancer centers around the United States.

## Supplementary Materials

Supplementary materials can be found at http://www.mdpi.com/1422-0067/19/5/1541/s1.

## Abbreviations

MCA	Mucinous colorectal adenocarcinoma
CRC	Colorectal cancer
AOM	Azoxymethane
DSS	Dextran sodium sulfate
RTK	Receptor tyrosine kinase
MUC2	Mucin 2
rhSCF	Recombinant human stem cell factor
Atoh1	Atonal homologue 1
AKT	Protein Kinase B
GSK3β	Glycogen synthase kinase 3β
CDX2	Caudal-related homeobox transcription factor
Spdef	SAM pointed domain containing ETS transcription factor
