# Supplementary material for: SCF/c-KIT Signaling Increased Mucin2 Production by Maintaining Atoh1 Expression in Mucinous Colorectal Adenocarcinoma
Source: Int J Mol Sci. 2018 May 22;19(5):1541. doi: 10.3390/ijms19051541 (PMC5983812; doi:10.3390/ijms19051541)
Supplement: Supplementary file 1 [file ijms-19-01541-s001.pdf]

Table S1. Human CRC samples

Type	All	non-MCA	MCA
	24	16 (66.7%)	8 (33.3%)
**Gender**			***P*=0.022**
Male	14	12	2
Female	10	4 (25%)	6 (75%)
**Tumor location**			***P*=0.667**
Right	10	7 (43.75%)	5 (52.5%)
Left	14	9	3
**Lymphatic invasion**			***P*=0.033**
No	10	10	1
Yes	14	6 (37.5%)	7 (87.5%)
**Distant metastasis**			***P*=0.362**
No	17	13	5
Yes	7	3 (18.75%)	3 (37.5%)

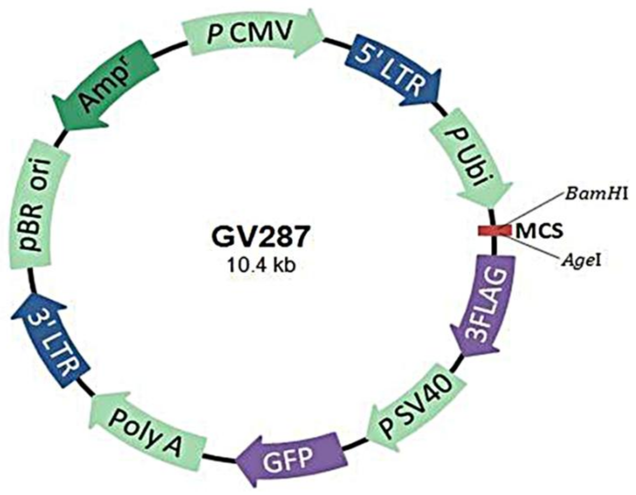

Figure S1. Scheme of lentivirus overexpressing *c-kit*.

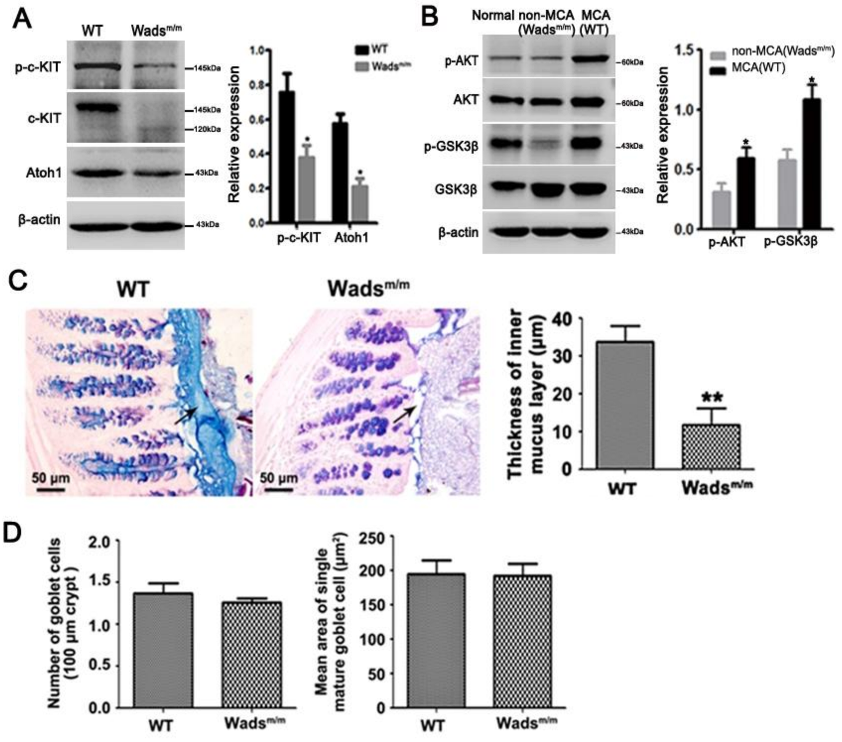

Figure S2. (**A**) The expressions of c-KIT, p-c-KIT and Atoh1 in normal mucosa were stronger in WT mice than those in Wads$^{m/m}$ mice, *P<0.05. (**B**) Compared with Wads$^{m/m}$-non-MCA tissues, the p-AKT and p-GSK3β were clearly increased in WT-MCA tissues, *P<0.05. (**C**) Alcian blue and PAS double staining showing inner mucus layer (arrow) in distal colon of WT and Wads$^{m/m}$ mice. The inner mucus layer in WT mice was continuous and densely compacted while it was incomplete and loose in Wads$^{m/m}$ mice. Significantly, the inner mucus layer was thinner in Wads$^{m/m}$ mice (n=5) than that in WT mice (n=5) , **P<0.01. (**D**) There was neither visible difference in goblet cell number between WT (n=5) and Wads$^{m/m}$ (n=5) mice, nor mean area of single goblet cell.
